# Efficacy of Curcumin with Iontophoretic Application on Paw Edema and Hematological Responses in Collagen-Induced Arthritis Rat Models

**DOI:** 10.1155/2020/4606520

**Published:** 2020-04-09

**Authors:** Ahmad Alghadir, Mohammad Miraj, Sharique Ali

**Affiliations:** ^1^Rehabilitation Research Chair, College of Applied Medical Sciences, King Saud University, Riyadh, Saudi Arabia; ^2^Department of Biotechnology, Saifia Science College, Bhopal, India

## Abstract

**Background:**

According to previous studies, oral administration of curcumin elucidates anti-inflammatory effect irrespective of its poor bioavailability. This study aims to measure the efficacy of lyophilized curcumin extracts with iontophoresis in arthritic rat models.

**Methods:**

Lyophilization and characterization of curcumin using the standard HPTLC method was carried out followed by induction of inflammatory arthritis in male albino rats. The animals were then treated with curcumin in three different forms, i.e., oral curcumin (OCU), oral curcumin with topical application (OCU + TOCU), and oral curcumin along with iontophoretically applied curcumin (OCU + IOCU). Various objective variables including body weight, paw edema, arthritic scores, and hematological and biochemical parameters, as well as histopathological examinations were conducted.

**Results:**

All the curcumin-treated groups showed significant alleviation of arthritic condition (*p*^*∗*^ < 0.05) when compared with arthritic controls. Group V (OCU + IOCU) demonstrated maximum therapeutic effect by restoring the body weight, decreasing the paw edema, and normalizing the Erythrocyte sedimentation rate and leukocyte count, when compared with other experimental rat groups (*p*^*∗∗*^ < 0.01).

**Conclusions:**

Iontophoretic administration of curcumin may ameliorate arthritic symptoms significantly, and the effect is assumed to be due to better penetration and enhanced bioavailability. Geriatrics patients are supposed to be benefited fairly by this technique.

## 1. Introduction

Rheumatoid arthritis (RA) is a chronic inflammatory systemic disorder that primarily affects smaller joints characterized by periods of severe pain, stiffness, and swelling and destruction of the bones and cartilage [1, 2]. Subsequently, other body parts also get involved including the eyes, lungs, heart, and blood vessels [2]. The annual incidence of RA is 3 in 10,000 people approximately, increasing with age and peaking between 35 and 50 years [3, 4]. The mode of management of RA, being a noncurable disease, is primarily palliative in nature. Different therapeutic regimens have been developed using various medicine systems worldwide to ameliorate the symptoms; however, it has limited treatment efficiency [5].

Among various therapeutics, allopathic treatment in the form of nonsteroidal anti-inflammatory drugs (NSAIDs) and disease-modifying anti-rheumatic drugs (DMARDs) is the mainstay of treatment universally [6]. These allopathic medications have severe side effects, and their prolonged use deteriorate the quality of life. The patients suffer from numerous side effects, commonly hyperacidity, swelling, stomach ulcer, gastrointestinal bleeding, perforation, and loss of appetite [3, 7].

Phytomedicines and chemical entities derived from plant sources have drawn global attention for their involvement in the treatment of various crippling disorders [7, 8]. One such compound is curcumin, which is the main active ingredient of *Curcuma longa* (turmeric) used as an additive and condiment in preparing Indian food since time immemorial [8]. The extracts from the roots of *Curcuma longa* have shown potential anti-inflammatory, antioxidant, chemopreventive, and chemotherapeutic activity in various models of cultured cells and animal studies [9]. Recently, curcumin has also been found to ameliorate a wide number of clinical conditions including cancer, inflammatory conditions, and digestive disorders [10, 11].

Surprisingly, there are only few scientific reports conducted for understanding the biomedical efficacy of this plant extract especially with reference to the treatment and cure of rheumatoid arthritis and similar other joint inflammatory disorders. A few literature argue that curcumin when administered orally reduces its bioavailability significantly [2, 3, 11, 12]. In physiotherapy, the iontophoretic method has been used for enhancing intradermal drug administration which was basically based on the electromagnetic principle which uses direct electrical current [12, 13].

Literature survey reveals no scientific reports for the assessment of anti-inflammatory and antiarthritic effects in rat models using iontophoresis for curcumin administration. The present study was undertaken to explore the possible anti-inflammatory and antiarthritic activity of the lyophilized extract of curcumin and its active ingredient curcumin using iontophoresis on certain arthritic and hematological markers.

## 2. Materials and Methods

The study was conducted taking into account three prominent sections. The first section comprised of collection, identification, characterization, and lyophilization of plant material, *Curcuma longa*. All the chemicals used in this study were of analytical grade and were obtained from verified sources.

### 2.1. Plant

#### 2.1.1. Procurement of Plant

The dried rhizomes of *C. longa* were purchased from an ayurvedic shop based in Bhopal and were identified, authenticated, and certified by a prominent ethnobotanist, Department of Botany, Saifia Science College Bhopal, with the voucher specimen referred as MS/DBT/CL/17.

#### 2.1.2. Preparation of the Extract

The lyophilized extract was prepared following the standard method as previously reported [14]. The method included the stages where the healthy, uninfected, and evenly colored dried rhizomes of *Curcuma longa* were boiled, dried, homogenized (Homogenizer model number 0210, Calton Instruments Company, New Delhi), and diluted with double glass distilled water (weight : volume = 1 : 10). The homogenized sample was then centrifuged (2000 ×g, at 25°C) for 10 minutes and filtered using Whatman filter paper (no. 1). The obtained supernatant was lyophilized in a freeze drier and then dried, which left a dark colored powder as residue [15].

#### 2.1.3. Pharmaceutical Analysis of Plant Extract

The lyophilized extract was then subjected to pharmaceutical analysis to ascertain its active ingredient by using the high-performance thin-layer chromatography (HPTLC) densitometry method reported earlier with some modifications [16]. The HPTLC method used an ultrasonicator (Enertech, Mumbai, India) for homogenizing of test and standard solutions followed by chromatographic separation of extracts of the *C. longa* performed on 20 cm × 10 cm aluminium-backed HPTLC plates coated with 200 pm layers of silica gel 60F254 (E. Merck, Darmstadt, Germany).

Chromatography was performed in Camag's twin-trough chamber where wavelength for detection of curcumin was evaluated from the complete UV spectrum of curcumin. Densitometric scanning was performed with a Camag TLC scanner 3 in the reflectance-absorbance mode at a wavelength (*λ*_max_) of 422 nm, under control of Camagwin CATS planar chromatography manager software (version 1.4.4) with the slit dimensions at 6 mm × 0.30 mm and scanning speed at 100 nm/s. This resulted in obtaining a chromatogram with peak of curcumin at Retardation factor (*R*_*f*_) of 0.47. Following the methods mentioned as previously reported [16, 17], the average value of the peak area was used for calculations after ensuring that the RSD was <2%. A calibration curve was plotted between increasing amounts of curcumin per spot and their peak area response which resulted in obtaining a straight line between 2.0 to 4.0 pg/spot.

### 2.2. Animals

Healthy adult Wistar strain albino rats (weighed 160 ± 20 g) were obtained from CPCSEA authorized animal facility. Subsequently, these animals were acclimatized for two weeks, under standard conditions of 28 ± 2°C and relative humidity (60 ± 10%) with a 12 : 12 hours light-dark cycle and were provided standard pellet diet and water *ad libitum* throughout the study to avoid any effects of starvation. No mortality was observed during the acclimatization period or during the entire study. Only healthy and active animals irrespective of sex were taken in the study. The animal care and handling were carried out in accordance with guidelines issued by CPCSEA.

#### 2.2.1. Toxicity Study

The measurement of LD_*50*_ for acute oral toxicity study was conducted as per the method reported earlier by Ecobichon [18, 19].

#### 2.2.2. Experimental Design

All the experimental animals were segregated into 5 groups with 15 animals in each described as Group I, fed with normal diet only and water *ad libitum*, and was called normal controls (NC) since they were not induced with arthritis. Group II was inoculated with type II collagen and was called as arthritic control (AC), while Group III was the arthritic rat group, which received treatment of oral curcumin (250 mg/kg body weight) for 45 days after development of arthritis. Group IV was the arthritic rat group which received treatment of oral curcumin (250 mg/kg body weight) along with plain topical application of curcumin on inflamed joint areas for 45 days after development of arthritis. Group V was fed with normal diet as well as received oral curcumin (250 mg/kg body weight) along with iontophoretic transdermal application of curcumin on inflamed joint areas for the same period of duration applied every alternative day. From each group, 5 rats were randomly selected and sacrificed at a regular time interval of 15, 30, and 45 days, for the evaluation of inflammatory and hematological parameters.

#### 2.2.3. Induction of Arthritis

Arthritis was induced through the standard procedure using Type II collagen obtained from the chicken tracheal cartilage purchased from Sigma-Aldrich (USA). In this method, the healthy, uninfected, acclimatized albino rats were immunized with II collagen, dissolved in 0.1 M acetic acid (2 mg/ml) at 4°C overnight, and emulsified with an equal volume of Complete Freund's adjuvant (CFA) (Sigma-Aldrich, USA) [20]. This was followed by each rat (*n* = 15) being injected intradermally with 4 mg/kg of collagen suspension at multiple areas around ankle joint and at the base of the tail. A booster dose injection was given after a week following the primary inoculation.

The development of rheumatic symptoms was observed following the second injection, paw edema being the first and most prominent visible signs of arthritic changes. The first set of readings for various evaluation parameters including paw edema was taken on the 15^th^ day. The second booster dose was administered 15 days after the first booster injection, i.e., on 22^nd^ day of experimentation. Following the protocol, the 1^st^ reading was taken on the 15^th^ day after immunization, and the subsequent readings were taken on the 30^th^day and the 45^th^ day of experimentation.

#### 2.2.4. Procedure for Iontophoretic Delivery

The iontophoretic delivery of lyophilized curcumin was followed as per the standard method reported earlier [21] which included two parts, i.e., preparation of animals and treatment of affected animals with curcumin. The iontophoretic drug delivery system used for the study (Model no.4201, HMS Corp., Tamil Nadu) comprised of four basic components including a power source with batteries, control circuitry called as electrical stimulator, electrodes, and the gauze pieces.

The preparation of the experimental animals before iontophoresis was carried out as per the method reported earlier where three days before iontophoresis, rats were anesthetized using intraperitoneal injection of ketamine methods reported earlier was carried (75 mg/kg) and xylazine (10 mg/kg). The delivery site was chosen, and furs from the dorsal area and hind legs were removed using scissors avoiding any abrasion or cuts at the selected sites. These exposed sites were cleaned using benzoin tincture and washed by water, and after drying, the site was wiped again with an alcohol swab and finally air-dried for iontophoresis as per the previously reported method [22].

For the iontophoretic procedure, one electrode was fixed on the exposed back of the inoculated animals using a surgical tape whereas the other electrode was fixed on the affected paw area near the ankle joint to complete the circuit. The dorsal electrode (on the back) was connected to the anode (positive pole), and the paw electrode was connected to the cathode (negative terminal). Between the electrode and the skin of the animals, a gauze piece moistened with 1 ml of 0.9% saline was filled under the dorsal electrode. The lyophilized curcumin dose for iontophoretic application was calculated to be 50 mg/ml, apart from 250 mg/kg of curcumin that was administered orally to the rats [22–25]. The pH of all solutions being applied iontophoretically was determined before dermal application using a microprocessor-based pH meter (Electronics India Ltd, India), and solutions used had a pH of between 5.5 and 6.5, which is suitable for epidermal application.

After following the abovementioned procedure, a gauze swab was taken and moistened with a solution of 50 mg/ml of curcumin dissolved in 0.2% DMSO. The gauze piece containing curcumin was then placed under the cathode electrode firmly, and then the electrode was placed on the inflamed paw area using adhesive tapes. The arthritic rats were treated with iontophoresis using direct current with an amplitude of 1 mA, applied for 10 minutes every alternate day. In a series of pilot experiments conducted earlier, the rats of the vehicle group were administered 0.9% saline solution instead of curcumin in their plantar region of the hind paw for iontophoretic transdermal delivery using similar conditions as that of curcumin treatment [21, 25]. It was found that there was no difference in the values of the parameters under investigation, i.e., general and hematological parameters were observed with that of noniontophoretic controls. Hence, the rats of the vehicle group were not kept for further experiments.

#### 2.2.5. Evaluation of Arthritic Scoring

The severity of arthritis in each paw was quantified by a clinical score measurement called as the arthritic score. The level of arthritic inflammation of each paw was graded from 0 to 4 as per the method reported earlier [20, 26] where 0 = absence of inflammation, 1 = slight inflammation, 2 = moderate inflammation, and 4 = marked inflammation. The maximum arthritic index (MAI) of four paws was summed for each rat and ranged from 0 to 16 where (0 indicates no disease and 16 refers to the highest possible score). The arthritic scores of the different curcumin-treated groups were compared with that of arthritic and normal controls for evaluation of the treatment efficacy by calculating the scores on the 15^th^, 30^th^, and 45^th^ day of experiments.

#### 2.2.6. Hematological Analysis

The collection of serum and other materials was carried out by sacrificing the animals by cervical dislocation on the 15^th^, 30^th^, and 45^th^ days of experimentation. Blood samples were collected by cardiac puncture, and a volume of 3-4 ml of blood was collected in a red color vacutainer tube which contained EDTA. The tubes were then centrifuged at 5000 rpm for 10 minutes at 4°C, and estimation of hemoglobin, ESR, RBC, and WBC as per methods reported earlier was carried out [26–30].

#### 2.2.7. Histopathological Assessment

After the collection of blood samples, the limbs of the animals were isolated and preserved for histopathological study. The histological samples comprised tissue samples of the ankle joint of normal, arthritic, and different forms of curcumin-treated arthritic rats which were immediately excised, fixed in buffered bouins, processed routinely for paraffin embedding, and sectioned at 5 microns. Sections were stained with hematoxylin and eosin (HE) and mounted with Canada balsam/DPX. Sections were examined by using an Olympus light microscope to detect histological damages and recoveries following the treatment, as per the method reported earlier [30, 31]. After being allowed to dry, sections were viewed using a Research trinocular microscope and photographed using a photo micrographic instrument of Nikon.

#### 2.2.8. Statistical Analysis

The data obtained were represented as Mean ± SEM. The statistical analysis was performed using the standard methods of inferential statistics [32] using GraphPad prism version 5 software program. Statistical significance of differences between the means of different experimental groups were assessed using the Wilcoxon *t*-test and one-way analysis of variance (ANOVA) followed by Dunett's multicomparison test. Level of significance was denoted by ^*∗*^*p* values < 0.05, ^*∗∗*^*p* < 0.01, and ^*∗∗∗*^*p* < 0.001.

## 3. Results

### 3.1. Analysis of the Lyophilized Extract of *Curcumin longa*

**Figure 1:**
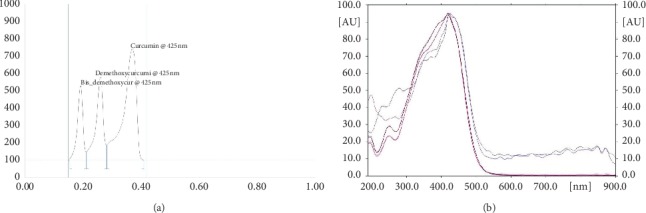
(a), (b) HPTLC Chromatograph of curcumin from the *C. longa* extract and absorption spectra of standard curcumin and the extract of *C. longa* taken on the CAMAG TLC Scanner III.

The HPTLC analysis of the lyophilized extract of *Curcumin longa* showed the presence of curcumin, the active ingredient, with an *R*_f_ value 0.47. The method for quantitative analysis of curcumin was validated with regard to its specificity, precision, accuracy, and linearity. The specificity of the method was ascertained by analyzing standard and samples. The spot for curcumin in the sample was confirmed by comparing the *R*_f_ value and the spectrum of the spot with that of standard, and the linearity is obtained which is shown in Figure 1. The average recovery percentage value was found to be 131.26 to 125.76 ± 1.33. Linearity was found over a concentration range of 1–5.0 ng/spot with a correlation coefficient (*r*) 0.98764 ± 2.20 (Figures 1(a) & 1(b)).

#### 3.1.1. Outcomes of the Toxicity Study and Dose Selection

The LD-50 calculated dose of curcumin was found to be 2500 ± 90 mg/kg body weight, whereas the dose selected for the treatment purpose of arthritic rats was 250 mg/kg body weight which is about ten times less than the LD-50 calculated dose [22–24].

#### 3.1.2. Effect of Treatment on the Body Weight

**Figure 2:**
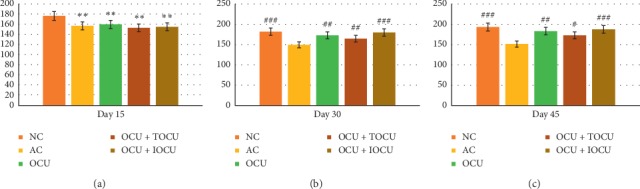
Changes in body weight (in grams, on the *Y*-axis) due to the effect of type II collagen-induced arthritis on the 15^th^, 30^th^, and 45^th^ days of the study; ^*∗*^ and # represent data compared against the normal group and arthritic control group, respectively, where ^*∗*^*p* < 0.05, ^*∗∗*^*p* < 0.01, and ^*∗∗∗*^*p* < 0.001 and ^#^*p* < 0.05, ^##^*p* < 0.01, and ^###^*p* < 0.001.

The general parameters were measured using body weight, paw edema, and arthritic score. Among the experiments, it was observed that while the body weight in the normal rat group increased from 168.4 ± 3.14 (gms) to 176.2 ± 2.87 (gms) over the first 15 days which further rose to 193.2 ± 4. 23 (gms) on the 45^th^ day, contrastingly, the experimental arthritic rat groups registered a decline in body weight from the baseline value of 165.8 ± 3.33 to 156.6 ± 3.56 (gms) in 15 days which further reduced to 151.4 ± 3.05 (gms) after 45 days of experimentation (*p* < 0.05). This indicated the effect of collagen inoculation in these rats, and iontophoretic application was completed (Figure 2).

On comparing the three experimental rat groups treated with curcumin in different forms, i.e., oral curcumin (OCU), oral with plain topical application of curcumin (TOCU), and oral with iontophoretically applied curcumin (IOCU) further referred to as group III, IV, and V, respectively, it was observed that significant reduction in body weight was found in all groups (*p* < 0.01). Oppositely, a significant gain in body weight (*p* < 0.01) was noticed in all these treatment groups at the end of the experiments on the dose administration.

#### 3.1.3. Implication of Treatment in Paw Edema

The first sensitive parameter to evaluate arthritic changes in the experimental animals was paw edema. The baseline, i.e., the paw edema, in normal control albino rats (NC) was 2.68 ± 0.65 (mm) which was found to increase to 4.70 ± 1.09 (mm) over 15 days of experimentation showing 67.85% increase in paw edema in comparison to controls. This increased to 5.16 ± 1.12 (mm) and 4.98 ± 1.15 (mm), respectively, after 30 and 45 days of experimentation, suggesting induction of collagen produced arthritic effects in the experimental rats (^###^*p* < 0.001).

**Figure 3:**
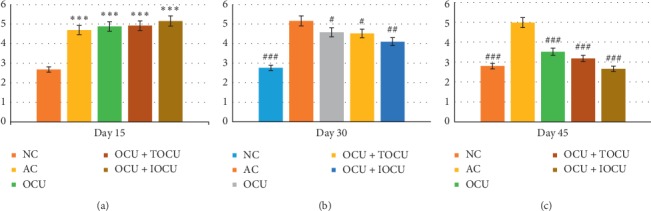
Changes in paw edema (in mm, on the *Y*-axis) following the treatment in type II collagen-induced arthritic rats on the 15^th^, 30^th^, and 45^th^ days of the study; ^*∗*^ and # represent data compared against the normal group and arthritic control group, respectively, where ^*∗*^*p* < 0.05, ^*∗∗*^*p* < 0.01, and ^*∗∗∗*^*p* < 0.001 and ^#^*p* < 0.05, ^##^*p* < 0.01, and ^###^*p* < 0.001.

On the other hand, the curcumin-treated rats registered a significant decrease across all groups at 30 days with paw edema readings been 4.58 ± 0.81 (mm), 4.52 ± 0.41 (mm), and 4.10 ± 0.60 (mm) in group III (OCU), group IV (OCU + TOCU), and group V(OCU + IOCU), respectively. At 45 days of experimentation, group V registered further decrease to 2.66 ± 0.40 (mm), bringing it closer to their prearthritic stages when the paw diameter measured was 2.54 ± 0.53 (mm) and, therefore, was most effective in the inhibition of paw edema consistently over the period of experiments from 0 to 45 days (^##^*p* < 0.01). Thus, it was observed that group V registered maximum decrease in paw edema (^###^*p* < 0.001) as compared to the other two treatment groups (Figure 3).

#### 3.1.4. Evaluation of Treatment Using Arthritic Score

**Figure 4:**
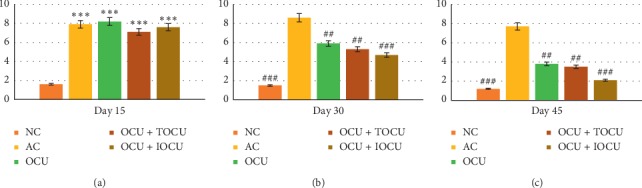
Showing comparative changes in arthritic score following the treatment in type II collagen-induced arthritic rats on the 15^th^, 30^th^, and 45^th^ days of the study; ^*∗*^ and # represent data compared against the normal group and arthritic control group, respectively, where ^*∗*^*p* < 0.05, ^*∗∗*^*p* < 0.01, and ^*∗∗∗*^*p* < 0.001 and ^#^*p* < 0.05, ^##^*p* < 0.01, and ^###^*p* < 0.001.

The gold standard to measure severity of arthritis was arthritic score. The mean arthritic score in the arthritic control group was 7.92 in comparison to the NC, where the score was 1.49 on the 15^th^ day of experimentation. The score rose significantly to 8.63 and 7.71 after 30 and 45 days, respectively (^##^*p* < 0.01). Similarly, an arthritic score of 3.87, 3.53, and 2.17 was measured on the 45^th^ day in three treatment groups, i.e., group III (OCU), group IV (OCU + TOCU) and group V (OCU + IOCU), respectively. Thus, it showed that group V was far better in inhibiting paw edema, redness, and restricted movement of the joints as compared to the other two experimental groups (^###^*p* < 0.001) (Figure 4).

#### 3.1.5. Effect of Treatment on Hematological Parameters

**Figure 5:**
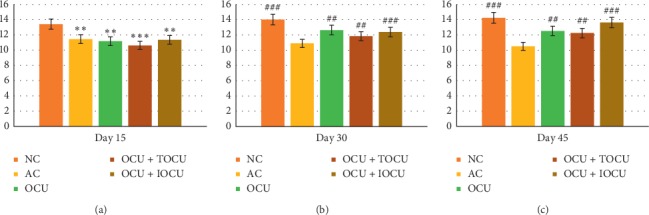
Changes in serum hemoglobin level (in gm/dl, on the *Y*-axis) following the treatment in type II collagen-induced arthritic rats on the 15^th^, 30^th^, and 45^th^ days of the study; ^*∗*^ and # represent data compared against the normal group and arthritic control group, respectively, where ^*∗*^*p* < 0.05, ^*∗∗*^*p* < 0.01, and ^*∗∗∗*^*p* < 0.001 and ^#^*p* < 0.05, ^##^*p* < 0.01, and ^###^*p* < 0.001.

It was observed that the baseline value of Hb in normal controls increased from 12.99 ± 1.72 (gm/dl) to 14.86 ± 0.94 (gm/dl), whereas in arthritic rats, it dropped significantly from 13.66 ± 1.20 to 10.50 ± 0.79 (gm/dl). For the same duration, after the initial dip on day 15 across all experimental groups, a significant rise was seen in Hb with 12.52 ± 0.83 (gm/dl), 12.24 ± 0.59 (gm/dl), and 13.62 ± 0.69 (gm/dl) in Group III, IV, and V, respectively. An increase of 20%, 15.25%, and 11.98% in Hb was observed in group V, IV, and III, respectively, in comparison to their well-matched arthritic controls where a fall by 23.13% was seen for the same time duration (^##^*p* < 0.01) (Figure 5).

**Figure 6:**
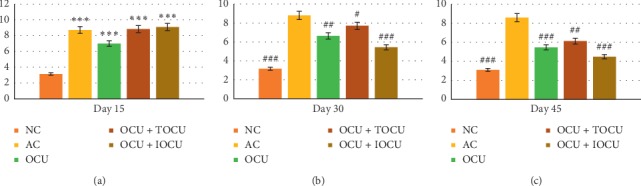
Changes in ESR (mm FHR, on the *Y*-axis) following the treatment in type II collagen-induced arthritic rats on the 15^th^, 30^th^, and 45^th^ days of the study; ^*∗*^ and # represent data compared against the normal group and arthritic control group, respectively, where ^*∗*^*p* < 0.05, ^*∗∗*^*p* < 0.01, and ^*∗∗∗*^*p* < 0.001 and ^#^*p* < 0.05, ^##^*p* < 0.01, and ^###^*p* < 0.001.

Similarly, on evaluating the ESR levels, arthritic controls showed significant increase of 151.74% depicting infection within the first 15 days, which increased further by 200% over 45 days (^###^*p* < 0.001). The ESR comparison between the experimental groups showed that group V recorded the most significant decrease bringing the ESR values from 9.1 ± 1.30 to 3.04 ± 0.42 (gm/dl) showing inhibition by 50.76% as compared to other two groups showing 22.22% and 30.77% in group III and IV, respectively, over the period of 45 days of experimentation (^###^*p* < 0.001) (Figure 6).

**Figure 7:**
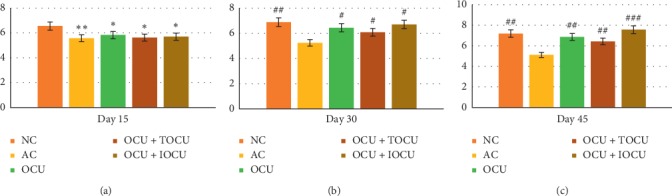
Changes in RBC (millions/mm^3^, on the *Y*-axis) following the treatment in type II collagen-induced arthritic rats on the 15^th^, 30^th^, and 45^th^ days of the study; *∗* and # represent data compared against the normal group and arthritic control group, respectively, where ^*∗*^*p* < 0.05, ^*∗∗*^*p* < 0.01, and ^*∗∗∗*^*p* < 0.001 and ^#^*p* < 0.05, ^##^*p* < 0.01, and ^###^*p* < 0.001.

On the other hand, RBC count also showed significant reduction (26.6%) in the arthritic controls as compared to the three experimental groups which recorded significant gain across all the three experimental groups with highest gain been found in group V (^###^*p* < 0.001) as compared to the other two experimental groups (groups IV and III) (^##^*p* < 0.01, Figure 7).

**Figure 8:**
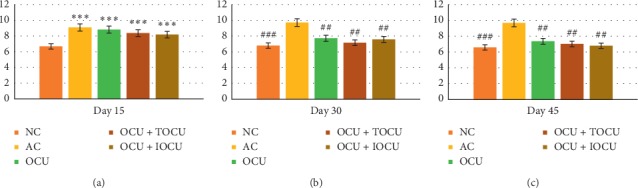
Changes in WBC (thousands/mm^3^, on the *Y*-axis) following the treatment in type II collagen-induced arthritic rats on the 15^th^, 30^th^, and 45^th^ days of the study; *∗* and # represent data compared against the normal group and arthritic control group, respectively, where ^*∗*^*p* < 0.05, ^*∗∗*^*p* < 0.01, and ^*∗∗∗*^*p* < 0.001 and ^#^*p* < 0.05, ^##^*p* < 0.01, and ^###^*p* < 0.001.

The leucocyte count registered a significant 37.11% increase as compared to the normal controls over the period of 45 days of experimentation. All the three experimental groups recorded significant fall in the leucocyte count with maximum fall been noticed in group V with 6.8 ± 0.89 (thousands/mm^3^) count as compared to the other two experimental groups been 7.36 ± 0.69 and 8.12 ± 1.21 (thousands/mm^3^), respectively (Figure 8).

#### 3.1.6. Histopathological Revelation of the Novel Therapy

**Figure 9:**
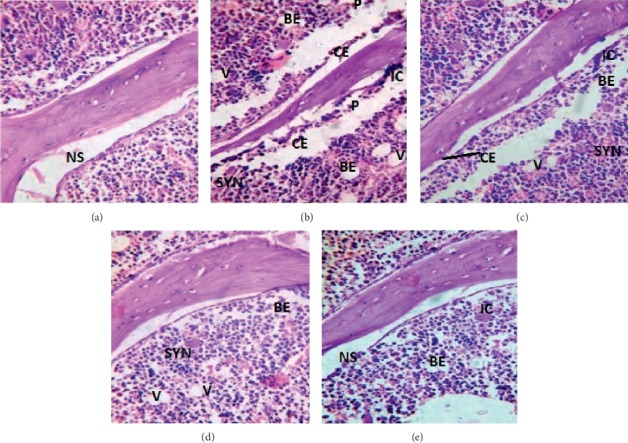
The histopathological changes of the ankle joint following the treatment in type II collagen-induced arthritic rats at the end of the study. (a) The normal architecture of the ankle joint in the NC group, with no pannus formation (P) and no bone erosion (BE), and the synovium (NS) was found to be normal. Additionally, cartilage erosion (CE) was absent, and the inflammatory cells (IC) were also not found, hence, showing the absence of arthritic condition. (b) In the AC group, pathological changes were observed as BE, CE, P, and IC were prominently seen, and synovial hyperplasia (SYN) and vacuole formation (V) were also noticed, which indicate the presence of arthritis. (c) In the OCU group, BE, CE, (V) SYN, and IC occurrences were reduced, and P was absent, so it may be inferred that oral curcumin had a minor role in improving the arthritic condition. (d) In the OCU + TOCU group, CE, P, and IC were completely absent, but moderate occurrence of SYN, BE, and V were seen; thus, it furthered the amelioration of arthritis. (e) In the OCU + IOCU group, significant alleviation in the arthritic condition was noticed as only mild BE and IC were present, thereby demonstrating the omission of arthritic condition and restoration of near normal synovium.

Histopathological examination of the photomicrographs reveals that an even surface of synovial lining with well-defined borders and a normal extracellular synovial matrix was observed in the NC group (Figure 9(a)). Contrastingly, a clear-cut erosion of the synovial membrane with uniform and compactly arranged synovial lining with significant damage, distortions, atrophy, and erosion of synovial lining was seen in the arthritic control group. Similar changes were also observed in the synovial matrix where near the damaged areas of the matrix, an increased proliferation of the synoviocytes was seen (Figure 9(b)).

The histological sections of the arthritic rats treated with curcumin in all the three experimental groups (group III, IV, and V) showed significant improvements in the damage caused to the synovial lining and the matrix, as shown in Figures 9(c)–9(e). In comparison to the histology of the synovium of the arthritic rats treated with oral curcumin with and without topical application (group IV and group III, respectively), the synovial lining had recovered considerably from the damage caused by arthritis, but there are still some abnormalities like irregular lining of the synovial membrane and scattered disfigured synoviocytes. Thus, there was no complete recovery of the ankle joints in the orally treated curcumin rats during the maximum treated period. On the other hand, iontophoretically applied curcumin for a period of 45 days showed significant recovery in the synovial architecture as compared to the arthritic controls.

## 4. Discussion

In the present study, the anti-inflammatory and antiarthritic effects of lyophilized curcumin extracts using iontophoresis as a treatment method in arthritic rat models were studied. During the analysis of the extract of dried rhizomes of *C. longa,* a higher content of curcumin, i.e., about 28.52%, was found (Figures 1(a) & 1(b)). Pothitirat and Gritsanapan reported that the quantity of curcumin content in the dried rhizome extracts of *C. longa* of various species growing in Thailand was in the higher range of 46.45% to 67.31% [33, 34]. A series of other studies reported the curcumin content in crude extracts (21.4% and 25.7%) as well as in essential oils depleted (21.69% and 34.50%), which is very close to our findings [35, 36].

In diseases such as rheumatoid arthritis (RA), fluctuations in body weight of patients are often observed; however, the reasons for the same are multifactorial [3, 4]. Rheumatoid cachexia is the increased catabolism which causes resting energy expenditure leading to weight loss and reduced lean body mass, especially if energy and protein requirements are not met, which had been found to be associated with RA [1, 2]. An association between weight loss and inflammatory joint disease has been recognized as early as in the 1940s when Hornell gave a detailed description of RA where weight loss was one of the key significant features [2, 3]. This explains the weight loss the animals suffered during the first 15 days of the study.

It has been reported that adjuvant-induced arthritis in rats is associated with cachexia because catabolic diseases are responsible for increasing the levels of circulating glucocorticoids which in turn causes anorexia, thereby causing weight loss in arthritic rats [2, 3, 6]. Our findings get considerable support from these earlier investigations, thereby suggesting that disease status progression and anti-inflammatory therapeutic response were indirectly linked to changes in body weight [10, 37, 38]. The study suggested that group V (OCU + IOCU) registered the maximum gain in body weight as compared to the other two groups over the 45 days of treatment (Figure 2) (^###^*p* < 0.001). This phenomenon might borrow explanation from the fact that curcumin treatment in both oral and iontophoretic form might have caused increased curcumin bioavailability in comparison to other groups. This might have restored the absorption capacity of the intestine and skin. The findings could be corroborated with recent reports where different plant extracts and their formulations were used in arthritic rat models causing restoration of intestinal absorption capacity, leading to weight loss reversal in arthritic rat models [37, 38].

The increase of paw thickness is an important clinical feature of arthritis. After inoculation, usually arthritis is completely developed between 13 and 18 days, and in the present study, the paw thickness registered a 67.85% increase on the 15^th^ day and 84.28% by the 30^th^ day from baseline reading. The rat model is a close resemblance to rheumatoid arthritis of human beings [26]. An early report by Yoshikawa et al. found that the hind paw volume and bone destruction were associated with the accumulation of leukocytes in the arthritic joint fluid and their secreted products and paw swelling were an important index for measuring antiarthritic activity of various drugs [39].

Correlating our findings with the severity of arthritis in the experimental animals, it was observed that the arthritic score of 7.71 was found in the type II collagen-induced arthritis as compared to the normal rats without arthritis scoring a 1.26 arthritic index. In the present study, it was found that rats treated orally and iontophoretically scored the least (2.17) followed by oral curcumin along with its local application (3.53). A significant difference was found between the arthritic score of oral along with iontophoresis application of curcumin in comparison with the other two curcumin-treated rat groups. However, on comparing between the two arthritic rat groups treated with oral curcumin plus topical applied curcumin and oral curcumin *per se*, no significant differences were observed. Thus, across the 45 days of treatment, the iontophoretically applied curcumin was better in inhibiting paw edema, redness, and restricted movement of the joints.

Hence, the data with the arthritic score index, paw edema, and body weight of the rats show interesting relationships. In the present study, increased severity of arthritis can be related to the degree of paw inflammation and joint stiffness in arthritic rats which can also be related to general body weight loss.

With reference to the various hematological parameters, the results showed significant reduction in the hemoglobin (Hb) and red blood corpuscles (RBC) levels across the study. Maximum reduction in RBC count was noticed on the 45^th^ day when it decreased from a control value of 7.19 ± 1.22 (in normal rats) to 5.11 ± 0.56 (millions/mm^3^) in arthritic rats, resulting in an 28.92% decrease in the RBC count and 26% reduction in hemoglobin concentration (Figures 5 and 7). This significant reduction in the RBC count and hemoglobin (Hb) concentration in the arthritis-induced rats could be attributed to two reasons: the first may be due to the premature destruction of red blood cells, and the second cause may be due to abnormal storage of iron in the reticuloendothelial system and synovial tissue, which might have caused low bone marrow iron availability due to decreased iron release by the mononuclear phagocyte system activation which had probably have resulted in ineffective erythropoiesis causing anemia.

On comparing the arthritic rats treated with a preselected dose of oral curcumin (250 mg/kg) body weight to the oral curcumin along with locally applied curcumin over the paw, it was found that no significant improvement was seen between the two groups (*p* < 0.05) in the initial 15 days of inducing arthritis. The period from the 15^th^ to 30^th^ days saw the maximum fall in the RBCs and hemoglobin (Hb) of rats of three experimental groups. On the 30^th^ day of experiment, the RBC and Hb count increased by 4.48% and 6.46%, respectively, in orally treated curcumin rats of group III. In its comparison, a rise of 4.07% and 5.27% was observed in RBC and Hb concentration, respectively, in arthritic rats treated with oral curcumin along locally applied curcumin (group IV). Similarly, the arthritic rats treated with oral curcumin *per se* along with its iontophoretic transdermal delivery increased the RBC count and Hb concentration by 4.59% and 6.99%, respectively. The data suggests that though improvements were observed in all the three experimental groups with maximum been seen in oral with iontophoretic curcumin (group V) followed by other 2 groups, the difference in the improvement between the groups III and IV lies statistically insignificant (*p* > 0.05).

In the present study, effects of oral curcumin in attenuating the damaging effect of induced arthritis may be attributed to the fact that certain inflammatory agents mimicking RA inflammatory metabolites released during adjuvant-induced arthritis models were counteracted by the oral curcumin in dosage of 250 mg/kg resulting which might have restored the gastrointestinal environment causing folic acid absorption and, hence, restoration of RBC count and the Hb concentration [35]. As compared to others in case of the iontophoretic transdermal delivery method, the improvements available were better because curcumin had better penetration. Since the curcumin induced into the body of the rats using iontophoresis did not had to go through first pass metabolism, the curcumin bio availability at the local level was probably more and believed to have caused the gastrointestinal alterations and probably improvements at the bone marrow level too.

On comparing the leucocyte count in the arthritic rats against normal controls, it was found that there was mild-to-moderate rise during the study period of 45 days. It appeared that the elevated level of WBCs in the induced arthritis as in the present case was due to the stimulation of the immune system against the invading antigens of arthritis. This might have led the gradual development of excessive leucocytes in arthritic rats. The restoration of leucocytes was maximum in iontophoretic transdermal delivery followed by oral along with plain topical application followed by oral curcumin alone. The difference between all the rat groups was found to be statistically significant. Our work was colinear with the previously reported work of Asmawi, which asserted that *Emblica officinalis* extract has an anti-inflammatory effect in carrageenan-induced arthritic rat models [40].

Thus, the change in the WBC count and its restoration can be explained in the way that induction of arthritis in the experimental rats was identified as a foreign body, leading to a cascade of reaction resulting in production of more WBCs to counteract the assault. This might justify the gradual development of excessive leucocytes in the peripheral blood of the arthritic rats. The results showed better efficacy of curcumin treatment using the iontophoretic drug delivery method which increased bioavailability and suppresses the migration of leucocytes into the inflamed area much better than the other conventional methods of herbal treatments. It was also found that oral curcumin along with plain local application was more effective in decreasing the WBC count as compared to oral curcumin alone. It could be due to the thin layer of the rat's skin that topical penetration was much greater causing curcumin to have reached the inflamed regions more effectively.

Additionally, the measurements of the Erythrocyte Sedimentation Rate (ESR), which is an indirect measurement of inflammation, is generally elevated in various stress conditions such as invasion/attack by pathogens on body, cell necrosis, and inflammation. In our study, it was found that ESR values increased significantly from 3.14 ± 0.42 to 8.72 ± 0.57 (mm FHR) showing 188.74% rise on the 15^th^ day, 191.39% on the 30^th^ day, and 184.10% on the 45^th^ day among the arthritic control rats. No significant difference was seen in first 30 days in the experimental groups; however, decline in ESR level was pronounced in the last 15 days of treatment of oral curcumin with iontophoretic application. This outcome gives an inference that bioavailability of curcumin was better than oral curcumin with/without plain local application of curcumin.

The histopathological assessment revealed that the uniform and compactly arranged normal synovial membrane was damaged and deranged with significant depletion of synoviocytes (Figure 9(a)). Group of rats receiving oral curcumin along with iontophoretic application showed the best recovery and restoration of the ankle joint. The synovial architecture of the ankle joint of the two groups, *viz*, oral curcumin with and without plain topical application, showed recovery from damage; however, complete restoration was not observed in either of them. The effective treatment among the animals receiving iontophoretic application of curcumin might be due to the reduced skin resistance, which is brought about by the electric potential gradient of the iontophoresis technique. The reduced skin resistance allows better penetration and, hence, increases the bioavailability. This in turn probably would have elucidated antiarthritic and anti-inflammatory actions on the antigens.

Contrastingly, in case of arthritic rats treated with oral curcumin alone, the curcumin molecules underwent gastrointestinal metabolism leading to poor bioavailability, and hence, lesser recovery was seen against the group treated with oral curcumin in conjunction with its iontophoretic application. Interestingly, lowering of skin resistance by iontophoretic application might have caused increased absorption of the drug at the transdermal level.

## 5. Conclusion

The outcomes of the presented study suggest that transdermal iontophoretic delivery may be a valuable tool for drug delivery in geriatric patients. The incompliance of the geriatric patients to the medications is well known; hence, this study opines that simultaneous use of transdermal curcumin via iontophoretic application along with oral curcumin is very effective in countering the arthritic symptoms. Nevertheless, further studies with larger sample size are recommended to understand the manifestations better and exploit the concept for clinical implications.

## Data Availability

The data used to support the findings of this study are available from the corresponding author upon request.
